# Uterine rupture disguised by urinary retention following a second trimester induced abortion: a case report

**DOI:** 10.1186/s12905-014-0159-9

**Published:** 2015-01-22

**Authors:** Qiaoying Jiang, Liwei Yang, Charles Ashley, Erin E Medlin, David M Kushner, Yanmei Zheng

**Affiliations:** Department of Obstetrics and Gynecology, Zhejiang Provincial People’s Hospital, Hangzhou, Zhejiang, People’s Republic of China; Department of Obstetrics and Gynecology, University of Wisconsin School of Medicine and Public Health, Madison, Wisconsin USA; Zhejiang Provincial People’s Hospital, NO.158 Shangtang Road, Hangzhou, Zhejiang Province 310014 China

**Keywords:** Uterine rupture, Urine retention, Misoprostol, Mifepristone, Laparoscopy

## Abstract

**Background:**

Uterine rupture classically presents with severe abdominal pain, loss of fetal station, vaginal bleeding, and shock.

**Case presentation:**

We present a case of uterine rupture presenting as significant urinary retention that occurred following a second trimester abortion induced with mifepristone and misoprostol. Uterine rupture was discovered unexpectedly on diagnostic laparoscopy. The uterine rupture was contained by dense adhesions between the omentum and bladder with the previous uterine cesarean hysterotomy scar.

**Conclusion:**

This case highlights the difficulties in diagnosis of abnormal placentation and an unusual presentation of uterine rupture. This case was managed successfully laparoscopically.

## Background

Second trimester pregnancy termination is a common gynecologic procedure and may be performed by surgical evacuation or induction of labor. Both surgical evacuation and induction of labor are safe and have rare complications including infection, blood loss, uterine rupture or perforation. Misoprostol is a synthetic prostaglandin E1 analogue which is widely used for induction of labor and is an effective agent for second trimester abortion [1]. Retrospective analysis has shown that mifepristone combined with a prostaglandin analogue in second trimester abortion is not associated with higher morbidity in women with a prior cesarean section [2]. A rare complication of second trimester induction of labor with a previous uterine scar is uterine rupture, which most commonly presents with abdominal pain, loss of fetal station, vaginal bleeding, and shock [3]. Here, we present an interesting case of uterine rupture during a second trimester abortion after oral administration of mifepristone and misoprostol. The patient presented with idiopathic fever and urinary retention, and the uterine rupture was diagnosed unexpectedly during exploratory laparoscopy.

## Case presentation

A 31-year-old multigravid woman with a history of a prior low transverse cesarean delivery presented to a family planning clinic at 16 weeks gestation for elective termination of pregnancy. Her obstetric history was significant for a prior cesarean section performed six years prior. Her past medical and surgical history was otherwise unremarkable. A transabdominal ultrasound performed on the day of admission confirmed a fetus of 16 week gestation with an anterior placenta. In order to further delineate the relationship between the uterine scar and the placenta, an abdominal MRI was performed. The MRI demonstrated an unclear uterine scar near the placental implantation site. The patient was counseled on risks associated with abnormal placentation and was offered uterine artery embolization, dilation and evacuation and hysterotomy. The patient, however, decided to proceed with induction of labor for termination. The termination of her pregnancy was approved by the ethics committee of Zhejiang Provincial People’s Hospital. A written informed consent was obtained from this patient before the procedure.

Termination was performed with a loading dose of 50 mg of oral mifepristone (RU486) followed by 25 mg administered every 12 hours until a total of 300 mg had been administered. Following completion of mifepristone, misoprostol was administered at a dose of 200 ug orally every hour. The patient received a total of 1200 ug of misoprostol. She had an unremarkable vaginal delivery with spontaneous delivery of the placenta. Vaginal bleeding was minimal. Four hours after the procedure the patient developed a fever of 38.5°C, but denied further symptoms. The patient’s temperature resolved spontaneously by the next morning without intervention. On post procedure day 1, the patient complained of abdominal distention and dysuria. A urethral catheter was inserted and 800 ml of clear urine was drained. A pelvic ultrasound was performed and demonstrated a 32 x 25 mm abnormal radiodensity at the anterior uterine wall with an unclear boundary at the anterior cervical wall, concerning for retained products of conception versus placenta accreta. Methotrexate was administered. Given the idiopathic urinary retention and abnormal findings on ultrasound, laparoscopic exploration was performed to evaluate for placental growth into in bladder.

**Figure 1:**
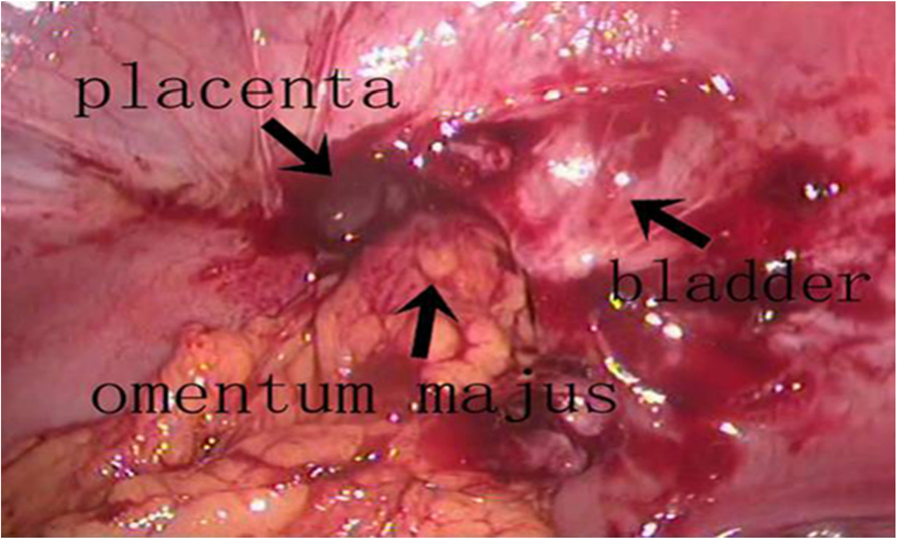
**A length of 1.5 cm anterior wall uterine rupture along the left previous scar was found.** The rupture was filled with small amounts of placenta and omentum majus.

**Figure 2:**
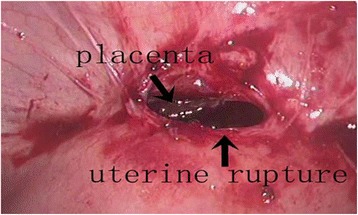
**A patch of placenta adhered to the rupture and lower uterine segment was observed when the omentum majus removed.**

**Figure 3:**
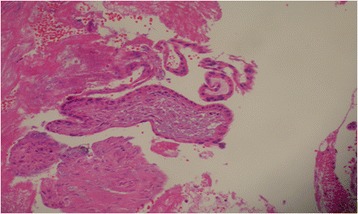
**Specimens sent for pathological examination were confirmed to be placenta.**

At the time of diagnostic laparoscopy, no hemoperitoneum was noted; however, there were significant adhesions between the omentum and anterior abdominal wall. Unexpectedly, a uterine rupture at the lower uterine segment was observed when the pelvic organs were restored to their normal anatomy. There was a 1.5 cm length anterior wall uterine rupture along the left portion of the previous scar (Figure 1). Moreover, a portion of the placenta was found to be adherent to the uterus at the site of the rupture when the omentum was removed (Figure 2). The bladder was firmly adherent to the uterine scar and rupture, and urology was consulted to assist in separating the bladder from the uterus. Dilatation and curettage was performed under direct laparoscopic visualization and the specimens were confirmed to be placenta by postoperative pathology examination (Figure 3). An ultrasound was performed to ensure no residual placental tissue was present. The uterine dehiscence was repaired laparoscopically. The entire procedure was performed using minimally invasive techniques.

## Discussion

Misoprostol has been widely used for medical abortion. Mifepristone has also been reported to be relatively safe and effective for use in the second trimester [4,5]. According to the recent clinical guidelines published by the Society of Family Planning (#2013-4), misoprostol is widely used as an off-label alternative or adjunct to osmotic dilators prior to dilation and evacuation in women desiring a second trimester pregnancy termination [6]. It is also safely used for labor-induction in mid second trimester abortions, even in women with prior uterine surgery [7]. A recent systematic review reported that the risk of uterine rupture in these women was less than 0.3% [3].

Uterine rupture is a rare but serious complication of second trimester induction of labor. Severe lower abdominal pain and shock caused by intraabdominal hemorrhage is the classic clinical presentation [8]. This patient, however, presented insidiously with urinary retention. The patient’s vital signs were clinically stable, and she reported no symptoms of abdominal pain. The ultrasound and MRI performed before the abortion raised concerns for abnormal placentation. The presentation of urinary retention raised concern for abnormal placentation with potential bladder involvement, although the patient had no evidence of hematuria, Methotrexate was administered timely to induce necrosis of trophocytes. Laparoscopic exploration was performed before dilatation and curettage to evaluate for bladder involvement. The uterine rupture was an unsuspected finding during the operation. The rupture was contained within the vesicouterine space, leaving only a small hole obscured by omental adhesions. Although it is unclear when the uterine scar dehisced, we suspect that this occurred prior to delivery given the dense adhesions, and further developed to uterine rupture with labor. Previous reports have noted similar findings of second trimester uterine rupture in the setting of abnormal placentation. This case was successfully managed laparoscopically. There is only one previous case report has described repair of uterine rupture with laparoscopic techniques [9].

## Conclusions

In summary, this is an unusual case of uterine rupture presenting with a primary complaint of urinary retention. This case highlights the difficulties in diagnosis of abnormal placentation and an unusual presentation of uterine rupture. Following a second trimester abortion, uterine rupture should be included in the differential diagnosis for patients presenting with urinary retention and continued vaginal bleeding. This case was successfully managed with laparoscopy. Minimally invasive surgery can be performed safelyy following a second trimester abortion and should be considered for management of complications following elective second trimester abortion.

### Consent

Written informed consent was obtained from the patient for publication of this case report and any accompanying images. A copy of the written consent is available for review by the editor of this journal.

## Abbreviation

MRI: Magnetic resonance imaging
